# Hectorite/Phenanthroline-Based Nanomaterial as Fluorescent Sensor for Zn Ion Detection: A Theoretical and Experimental Study

**DOI:** 10.3390/nano14100880

**Published:** 2024-05-19

**Authors:** Marina Massaro, Ana Borrego-Sánchez, César Viseras-Iborra, Giuseppe Cinà, Fátima García-Villén, Leonarda F. Liotta, Alberto Lopez Galindo, Carlos Pimentel, Claro Ignacio Sainz-Díaz, Rita Sánchez-Espejo, Serena Riela

**Affiliations:** 1 Dipartimento di Scienze e Tecnologie Biologiche, Chimiche e Farmaceutiche (STEBICEF), Università di Palermo, Viale delle Scienze, Ed. 17, 90128 Palermo, Italy; marina.massaro@unipa.it (M.M.); giuseppe.cina05@unipa.it (G.C.); 2 Instituto de Ciencia Molecular, Universitat de València, Carrer del Catedrátic José Beltrán Martinez 2, 46980 Paterna, Spain; ana.maria.borrego@uv.es; 3 Department of Pharmacy and Pharmaceutical Technology, Faculty of Pharmacy, Campus Universitario de Cartuja, University of Granada, 18071 Granada, Spain; fgarvillen@ugr.es (F.G.-V.); ritamsanchez@ugr.es (R.S.-E.); 4 Andalusian Institute of Earth Sciences, Consejo Superior de Investigaciones Científicas-University of Granada (CSIC-UGR), Av.da de las Palmeras 4, 18100 Armilla, Spain; alberto.lopez@csic.es (A.L.G.); ci.sainz@csic.es (C.I.S.-D.); 5 Istituto per lo Studio dei Materiali Nanostrutturati-Consiglio Nazionale delle Ricerche (ISMN-CNR), Via Ugo La Malfa 153, 90146 Palermo, Italy; leonardafrancesca.liotta@cnr.it; 6 Departamento de Mineralogía y Petrología, Facultad de Ciencias Geológicas, Universidad Complutense de Madrid, C/José Antonio Novais, 12, 28040 Madrid, Spain; cpimentelguerra@geo.ucm.es; 7 Dipartimento di Scienze Chimiche (DSC), Università di Catania, Viale Andrea Doria 6, 95125 Catania, Italy

**Keywords:** hectorite, phenanthroline, fluorescent sensor, Zn ion detection

## Abstract

The development of fluorescent materials that can act as sensors for the determination of metal ions in biological fluids is important since they show, among others, high sensitivity and specificity. However, most of the molecules that are used for these purposes possess a very low solubility in aqueous media, and, thus, it is necessary to adopt some derivation strategies. Clay minerals, for example, hectorite, as natural materials, are biocompatible and available in large amounts at a very low cost that have been extensively used as carrier systems for the delivery of different hydrophobic species. In the present work, we report the synthesis and characterization of a hectorite/phenanthroline nanomaterial as a potential fluorescent sensor for Zn ion detection in water. The interaction of phenanthroline with the Ht interlaminar space was thoroughly investigated, via both theoretical and experimental studies (i.e., thermogravimetry, FT-IR, UV-vis and fluorescence spectroscopies and XRD measurements), while its morphology was imaged by scanning electron microscopy. Afterwards, the possibility to use it as sensor for the detection of Zn^2+^ ions, in comparison to other metal ions, was investigated through fluorescent measurements, and the stability of the solid Ht/Phe/Zn complex was assessed by different experimental and theoretical measurements.

## 1. Introduction

In the last year, the development of fluorescent sensors has gained considerable attention since they possess advantageous properties in comparison to other sensing methods, for example, spectrophotometry [1,2,3]. Fluorescent sensors are widely utilized for the detection of biomolecules or metal ions due to their high sensitivity, high specificity, immunity to light scattering, and ease of operation [4,5,6,7]. Among the different fluorescent molecules that can be used for metal ion detection, 1,10-Phenanthroline (Phe) is the most used as a chelant ligand [8]. Due to its planarity and rigidity structure features, phenanthroline shows excellent spectral properties, such as high-fluorescence quantum yield, high molar extinction coefficient, and visible-light excitation. To expand the application field of phenanthroline as a sensor, different derivatization strategies have been explored to obtain water-soluble phenanthroline derivatives [9].

Clay minerals are natural materials, biocompatible, and available in large amounts at a very low cost [10]. Due to their physico-chemical properties, clay minerals have been used to accommodate different organic molecules [11], such as drugs [12], dyes, and fluorophore [13]. Among the different clay minerals used to load organic molecules, those belonging to smectite groups show interesting properties [14]. These are layered clay minerals possessing large surface area, swelling in water, thermal and chemical stabilities, and the ability to accommodate a wide variety of guest species, from ions/molecules to polymers/nanoparticles [15,16]. The driving forces for the loading of organic molecules are cation exchange, ion–dipole interactions, and hydrophobic effects [17]. 

Hectorite (Ht) is a natural layered magnesium–lithium silicate belonging to the smectite group, composed of different layers of Si−O−Mg(Li)−O−Si−, with hydrated cations (e.g., Na^+^, Li^+^) in the interlayer space and an ideal chemical formula of Na_0.3_Mg_2.7_Li_0.3_Si_4_O_10_(OH)_2_ [18]. Because of its structure and chemical composition, hectorite possesses negative charges on the basal faces (mainly constituted by siloxane groups) and pH-dependent charge on the edges.

Herein, we report the intercalation of phenanthroline molecules into hectorite for the development of a fluorescent sensor for Zn ion detection in a water solution.

Zn^2+^, indeed, is involved in different biological processes. For example, its excess in the body might be correlated to Alzheimer’s disease and Parkinson’s disease [19], while alopecia is associated with a lack of these ions [20]. Thus, the development of selective methods to detect Zn^2+^ ions in aqueous solution is very important.

The Ht/Phe nanomaterial was synthetized and thoroughly characterized from a physico-chemical point of view and via DFT measurements. Then, the possibility to use it as a sensor for the detection of Zn^2+^ ions was investigated through fluorescent measurements, and the stability of the solid Ht/Zn/Phe complex was assessed using different experimental and theoretical measurements.

## 2. Materials and Methods

All reagents needed were purchased from Merck (Milan, Italy) and used without further purification. Hectorite was kindly gifted by Tolsa Group Inc. (Madrid, Spain).

FT-IR spectra (KBr) were recorded using an Agilent Technologies Cary 630 FT-IR spectrometer (Agilent Technologies, Santa Clara, CA, USA). Specimens for these measurements were prepared by mixing 5 mg of the sample powder with 100 mg of KBr.

The thermogravimetric analysis (TGA) of the material was performed in a TGA/DSC1 STAR System (Mettler Toledo Inc., Madrid, Spain). The sample (15 mg) was subjected to a pre-treatment in air flow (30 mL/min) from 25 °C to 100 °C, with a heating rate of 10 °C/min and holding time at 100 °C for 30 min, in order to remove any eventual physisorbed water. Then, the temperature was increased from 100 to 1000 °C under air flow (30 mL/min), and the weight loss occurring during this step was considered when calculating the organic weight content of the Ht-based nanomaterial.

UV-vis measurements were made with a Beckmann DU 650 spectrometer (Beckman Coulter, Inc., Brea, CA, USA).

Scanning electron microscopy (SEM) images were acquired with a GEMINI (FESEM) (CARL Zeiss, Oberkochen, Germany) coupled to an EDX microanalyzer (Oxford Instruments, Abingdon, UK). Samples were dried at 40 °C for a minimum of 48 h. Then, powdery samples were mounted over standard aluminum stubs and coated with carbon. Textural microphotographs were obtained at 3 kV, while EDX was acquired at 15 kV. In both cases, immersion lens detector was used (InLens, Zeiss, Oberkochen, Germany).

X-ray powder diffraction (XRPD) analysis was carried out using a diffractometer (X’Pert Pro model, Malven Panalytical, Madrid, Spain) equipped with a solid-state detector (X’Celerator) and a spinning sample holder. The diffractogram patterns were recorded using random oriented mounts with CuKα radiation, operating at 45 kV and 40 mA, in a range 4–60° 2θ.

Fluorescence measurements were performed with a JASCO FP8300 spectrofluorometer (JASCO, Cremella (LC), Italy) with excitation and emission slits set at 5 nm, and spectra were acquired in wavelength interval ranging between 300 and 700 nm.

### 2.1. Synthesis of Ht/Phe Nanomaterial

To an aqueous phenanthroline solution (1 × 10^−2^ M, 20 mL), 200 mg of pristine Ht were added. The obtained dispersion was stirred for 18 h at room temperature. After this time, the solvent was filtered off, and the obtained powder was washed several times with water and dried at 60 °C overnight.

### 2.2. Synthesis of Ht/Zn/Phe Nanomaterial

To an aqueous ZnCl_2_ solution (0.5 M, 5 mL), 200 mg of Ht were added, and the obtained dispersion was left to stir at room temperature overnight. Afterwards, the dispersion was centrifuged, and the obtained solid was washed several times with water and dried at 60 °C overnight. After this time, the Ht/Zn nanomaterial obtained was resuspended in 20 mL of an aqueous phenanthroline solution (1 × 10^−2^ M), obtaining a dispersion that was left to stir at room temperature overnight. The obtained powder was centrifuged and washed with water to remove any unreacted phenanthroline molecules and finally dried at 60 °C.

### 2.3. Models

The phenanthroline molecular structure was taken from a previous work [21]. A unit cell model of Ht was created from previous crystallographic data, with Na^+^ and Zn^2+^ as interlayer cations [22]. This model was simplified by substituting the F^−^ anions with OH^−^ ones. A 2 × 2 × 1 supercell of this model was created, taking into account the size of the phenanthroline molecule. The chemical composition was slightly adjusted to avoid partial atomic occupancies with formula Na_2_(Mg_22_Li_2_)Si_32_O_80_(OH)_16_ (NaHt), and Zn(Mg_22_Li_2_)Si_32_O_80_(OH)_16_ (ZnHt). Further, 3D periodic boundary conditions were applied. Six water molecules were intercalated in the interlayer space of these supercells for hydrating the Na^+^ and Zn^2+^ cations. The adsorption of phenanthroline was studied on these two hectorite structures. The organic molecule was placed in the interlayer space of both hectorites.

### 2.4. Computational Methodology

Quantum mechanical calculations based on Density Functional Theory (DFT) were performed using the CASTEP program within BIOVIA Materials Studio software (v 22.1) [23]. The generalized gradient approximation (GGA) and the Perdew–Burke–Ernzerhof (PBE) functionals were used. On-the-fly-generated (OTFG) ultrasoft pseudopotentials were also used, with Koelling–Harmon relativistic treatment. An energy cutoff of 570 eV was chosen, and the effect of the Tkatchenko–Scheffler dispersion corrections was included. Furthermore, the calculations were performed applying periodical boundary conditions [23]. This computational approach was previously suitable for the study of another drug–clay system [24]. In this work, the described methodology was used to optimize the geometry of the models of the sodium and zinc hectorites and the adsorption complexes of phenanthroline with both hectorites.

### 2.5. Fluorescence Titration for Metal Ion detection

To a dispersion of Ht/Phe nanomaterial in water (0.05 mg/mL), 750 μL of different salts solutions (1 × 10^−4^ M) was added, to a final volume of 2 mL. The obtained dispersions were degassed for 10 min under Ar flow. Excitation and emission slits were set at 2.5 nm, and spectra were acquired in a wavelength interval ranging between 270 and 700 nm.

### 2.6. Fluorescence Titration for Zn^2+^ Ion detection

To a dispersion of Ht/Phe nanomaterial in water (0.05 mg/mL), increasing volumes (0–1 mL) of an aqueous salt solutions (1 × 10^−4^ M) were added. The obtained dispersions were degassed for 10 min under Ar flow. Excitation and emission slits were set at 2.5 nm, and spectra were acquired in a wavelength interval ranging between 270 and 700 nm.

### 2.7. Paper Strips

Filter papers were imbibed with 100 μL of Ht/Phe aqueous dispersion (1 mg mL^−1^) and dried at 60 °C for 10 min. Afterwards, 100 mL of salt solution (0.01 M) was added, and the obtained strips were dried at room temperature. Colors of paper strips were determined under 265 nm ultraviolet light.

## 3. Results and Discussion

The interaction of Phe with Ht was studied using two different approaches: (i) quantum mechanics calculations and (ii) experimentally, from a physico-chemical point of view. 

### 3.1. Theoretical Study of the Adsorption of Phenanthroline Molecule in Sodium Hectorite

**Figure 1:**
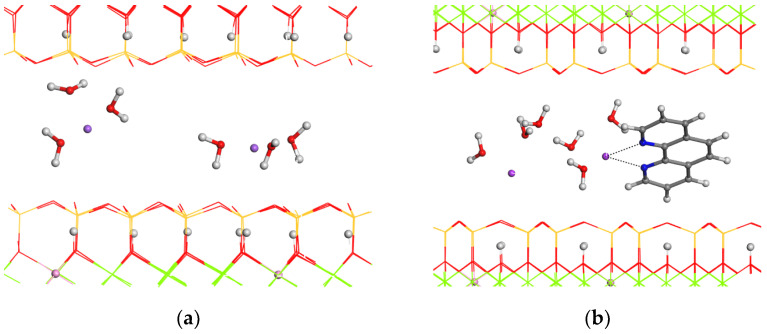
Optimized structures of (**a**) NaHt and (**b**) NaHt/Phe. The O, H, N, Mg, Si, Li, Na, and C atoms are depicted in red, white, blue, green, yellow, pink, purple, and grey colors, respectively. The octahedral Li atoms, the H atoms and interlayer atoms are highlighted as balls.

A 2 × 2 × 1 supercell of a hydrated hectorite with two sodium cations in the interlayer space (NaHt) was optimized (Figure 1a). After NaHt geometric optimization, the water molecules solvated the Na^+^ cations, and the d(001) spacing was 12.23 Å according to the experimental value. Afterwards, a phenanthroline molecule was added in the interlayer space, and the whole structure of the NaHt/Phe complex was also optimized (Figure 1b). 

In all models, the OH groups of Ht are oriented perpendicularly to the 001 plane. This orientation is not altered with the adsorption of Phe. In the NaHt/Phe complex, the phenanthroline molecule adopts an orientation non-parallel to the 001 plane, being d(001) = 17.0 Å, close to the experimental value. Nevertheless, this value is smaller than the experimental one due to the lower water content of this model than in the experiment. By comparing energies (i.e., product minus reactive), we can conclude that the adsorption of phenanthroline is energetically favorable for hectorite. The adsorption energy of phenanthroline intercalated in the interlayer space of Na-hectorite is −2.79 eV. 

### 3.2. Synthesis and Characterization of Hectorite/Phenanthroline-Based Sensor

The loading of phenanthroline (Phe) onto Ht was accomplished as follows: A concentrated phenanthroline solution in water (1 × 10^−2^ M) was added to an aqueous dispersion of Ht. The dispersion was left to stir at room temperature for 24 h. After loading, the Ht/Phe nanomaterial was washed several times with water to remove free molecules. After work-up, the percent loading of Phe in the final Ht/Phe nanomaterials was as large as 9 wt%, as estimated by thermogravimetric analysis (TGA). The nanomaterial obtained was thoroughly characterized by TGA, FT-IR, and UV-vis spectroscopies and XRD measurements. 

**Figure 2:**
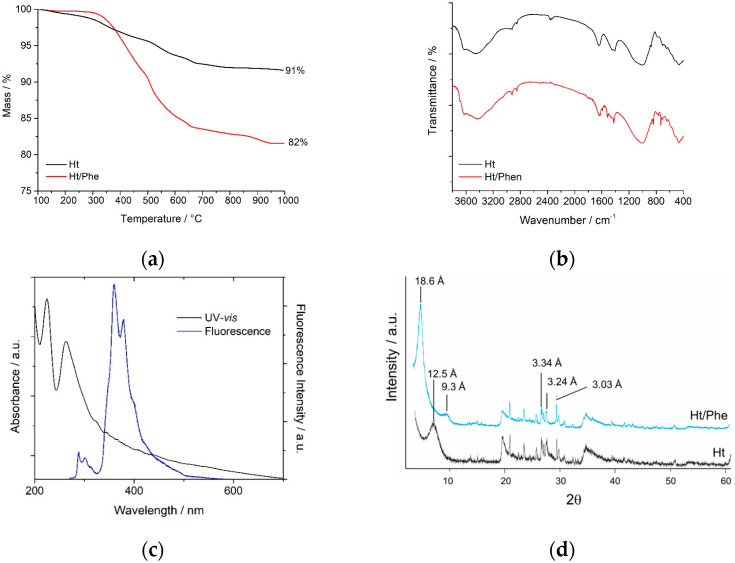
(**a**) Thermogravimetric curves, (**b**) FT-IR spectra, (**c**) UV-vis and fluorescence spectra and (**d**) XRD patterns of Ht, and Ht/Phen nanomaterial.

In Figure 2a, the thermogravimetric curves of the Ht/Phe nanomaterial and that of pristine Ht for comparison are reported. It is possible to observe that the synthetized nanomaterial, similar to Ht, shows a net weight loss in the range ~300–700 °C, corresponding to the loss of physisorbed and hydration water present in the lamellar structure of the material, with some additional mass losses attributable to the degradation and volatilization of the organic portion, reaching, at 1000 °C, a constant weight equal to ca. 82 wt%. Conversely, for the pristine Ht, the removal of hydration water bound in the structure occurs with a gradual and continuous decomposition process, up to a value of 91 wt%.

On the basis of the thermogravimetric curves, the loading of phenanthroline into Ht was calculated as reported above. The FT-IR spectra of the Ht/Phen nanomaterial and, for comparison, that of pristine Ht are shown in Figure 2b. The FT-IR spectrum of the Ht/Phe nanomaterial showed the typical vibration bands of hectorite with the presence of additional bands related to the presence of the organic molecule. In particular, the stretching vibration peaks of C=N and C=C double bonds in a range 1600–1500 cm^−1^ and the out-plane bending vibration peak of C-H bond at 735 cm^−1^ are clearly observable, further corroborating the presence of phenanthroline in the clay.

UV–vis and fluorescence spectroscopies further confirmed the presence of phenanthroline molecules into Ht. As can be observed in Figure 2c, the aqueous dispersion of the nanomaterial exhibits characteristic absorption bands at ca 225 and 260 nm, attributable to π → π* transitions inside the Phe ring, whereas the emission spectrum shows a typical emission band at 360 nm, originating from the π → π* singlet excited state inside the Phe ring [25].

To further prove the intercalation of Phe into the lamellar structure of Ht, some XRD measurements were performed (Figure 2d).

The original sample, Ht, is mainly made up of hectorite, a trioctahedral smectite, where part of the magnesium of the octahedral sheet is substituted by lithium. Lesser amounts of quartz (3.34 Å), K-feldspars (3.24 Å), and calcite (3.03 Å) were also identified by X-ray diffraction.

The d(001) spacing of nontronite is 12.5 Å, indicating the dominance of a monovalent cation (Na) in the interlayer.

When phenanthroline is added to Ht, the d_001_ basal spacing of hectorite increases to around 18.6 Å according to the intercalation of an organic molecule. Due to the increased order in the stack, its harmonic (002) reflection appears to be 9.3 Å. The addition of phenanthroline does not change the diffraction peaks of the accessory minerals, and their main reflections are still present.

**Figure 3:**
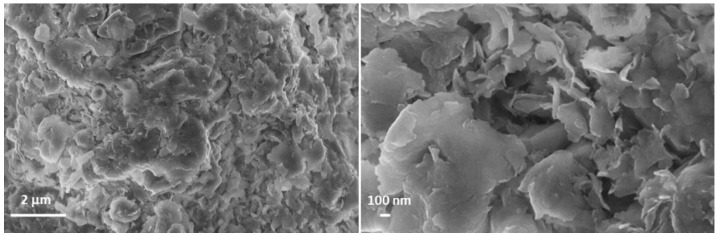
SEM images of Ht/Phen nanomaterial.

The morphology of the Ht/Phen nanomaterial was investigated by scanning electron microscopy (SEM). SEM images (Figure 3) of the Ht/Phe nanomaterial showed that, after Phe loading, the morphology of Ht was preserved. Similar to pristine Ht, indeed, the nanomaterial showed a compact layered structure, where the Ht nano-disks were blocky in shape [26].

### 3.3. Metal Ion Complexation 

#### 3.3.1. Selectivity

**Figure 4:**
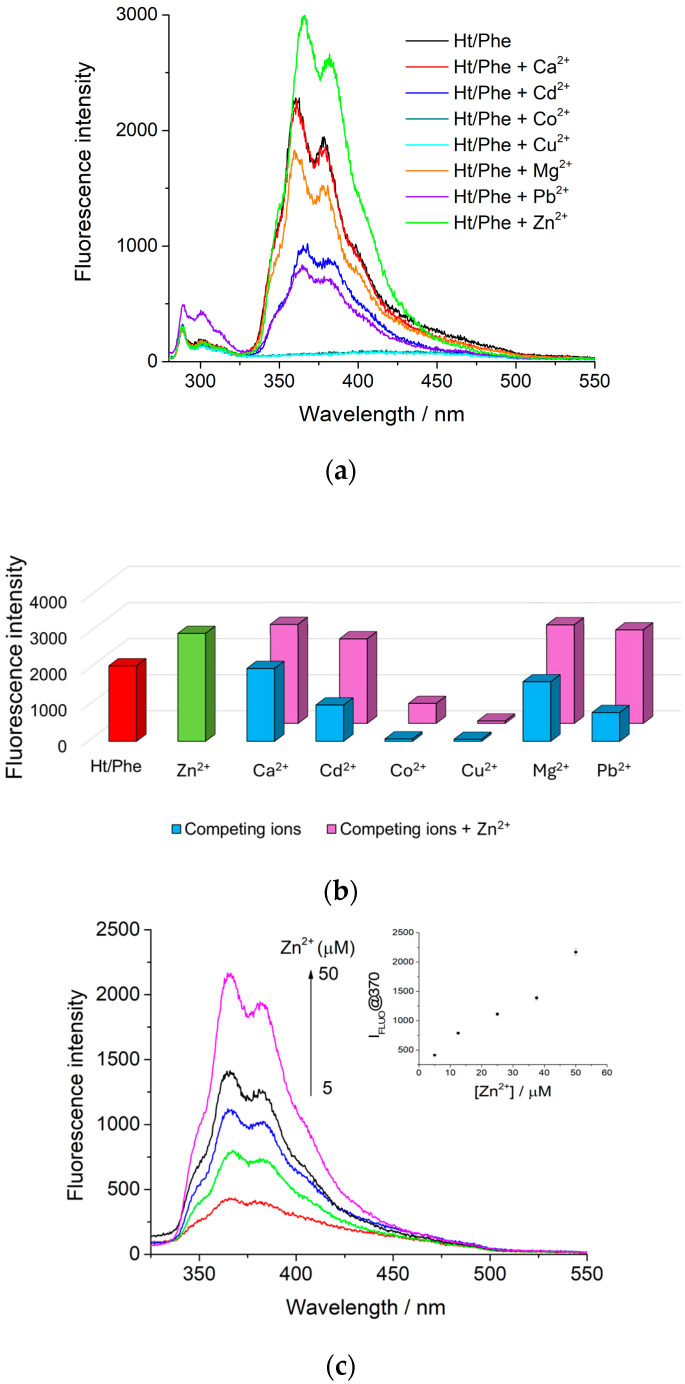
(**a**) Changes in fluorescence emission spectra (λ_ex_ = 260 nm) of Ht/Phe (0.05 mg mL^−1^) with addition of different metal ions (37.5 μM) in water; (**b**) fluorescence intensity of Ht/Phe nanomaterial in the absence (red bar) and in the presence of different metal cations (37.5 μM) with (pink bars) and without (blue bars) the addition of Zn^2+^ ions (37.5 μM) in water, in green bar is reported the Ht/Phe fluorescence response to Zn^2+^ addition; (**c**) fluorescence spectra of Ht/Phe nanomaterial upon addition of different concentration of Zn^2+^ ions (5–50 μM).

To validate the selectivity of the synthetized Ht/Phe probe for Zn^2+^ ions, its fluorescence response behavior (37.5 μM) was investigated through the addition of various metal ions (Co^2+^, Ca^2+^, Mg^2+^, Zn^2+^, Cd^2+^, and Pb^2+^) in water, with excitation at 260 nm. As shown in Figure 4a, the addition of Co^2+^ and Cu^2+^ ions led to a quenching of phenathroline fluorescence, while the addition of Ca^2+^ and Mg^2+^ did not show any variation in the emission properties of the Ht/Phe nanomaterial. The introduction of Pb^2+^ and Cd^2+^ ions led to a significant fluorescence emission reduction, without any shift in the emission band of Ht/Phe.

Interestingly, Zn^2+^ ions increase the fluorescence emission of Ht/Phe, with a bathochromic shift in the emission band of ca. 10 nm, from 360 to ca. 370 nm, indicating that the Ht/Phe nanomaterial synthetized could be useful for the determination of Zn^2+^ ions in aqueous solution in a similar way to the free phenanthroline (Figure S1). To assess the specific selectivity of the Ht/Phe nanomaterial toward Zn^2+^ ions in the presence of other coexisting competitive metal ions, a Zn^2+^ solution (37.5 μM) was added to dispersions of Ht/Phe and other metals (blue bars in Figure 4b). As is possible to observe in Figure 4b, the addition of Zn^2+^ resulted in an enhancement in the emission intensity of the Ht/Phe nanomaterial in almost all cases investigated (pink bars). These findings further suggest that the developed Ht/Phe nanomaterial shows selectivity for the detection of Zn^2+^ and that other metal ions do not interfere with its binding affinity with Zn^2+^.

#### 3.3.2. Fluorescence Response to Zn^2+^ Ions and Reversibility Studies

Thus, the variation in the Ht/Phe emission spectrum upon the addition of increasing the concentration of Zn^2+^ ions was investigated. As is possible to note from Figure 4c, by increasing the concentration of Zn^2+^ ions, an increase in the emission band at 370 nm of the Ht/Phe nanomaterial occurs, indicating the formation of a coordination complex between Zn^2+^ and Ht/Phe. From the linear fitting of the experimental data, it was possible to calculate the limit of detection (*LOD*) by applying the following equation:

(1) $$LOD=3\left(\frac{\sigma }{k}\right)$$

where *σ* and *k* are the standard error of the intercept and the slope of the regression line (insert in Figure 4c), respectively. In this work, the LOD value is 5.2 μM, in line with that reported in the literature (Table 1), with the advantage of using a low-cost, natural, and biocompatible nanomaterial, such as hectorite, that can be recovered by centrifugation, without long and tedious purification methods. Furthermore, considering that the human blood plasma typically presents Zn^2+^ ions in a concentration range of 6–12 μM [27], the developed nanomaterial could be successfully used for biological purposes.

**Figure 5:**
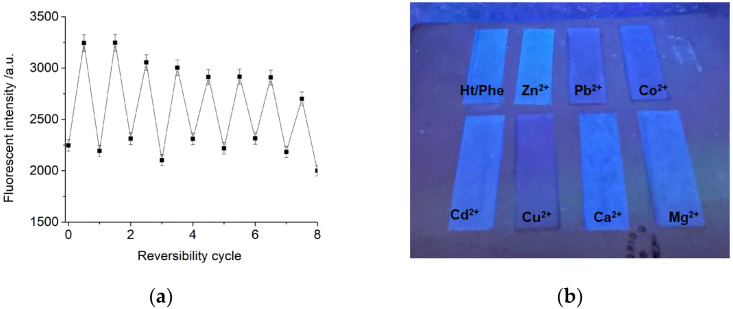
(**a**) Reversibility of Ht/Zn nanomaterial (0.05 mg mL^−1^) upon sequentially adding Zn^2+^ and EDTA, *λ*_ex_ = 260 nm (error bars represent SD (*n* = 3)); (**b**) photographs of test strips for detecting various cations under illumination at 265 nm.

The fluorescence increase in the Ht/Phe nanomaterial upon the addition of Zn^2+^ ions was reversible, as testified by the reversibility performed in the presence of EDTA. Upon subsequent additions of Zn^2+^ ions and EDTA, a turn “off-on-off” of the fluorescence signal was observed, for at least eight cycles, with low fluorescence loss (Figure 5a).

#### 3.3.3. Strip Test

Finally, to test the practical application of the Ht/Phe nanomaterial, strip tests on filter paper were carried out. To do this, 100 μL of an aqueous dispersion of the Ht/Phe nanomaterial (1 mg mL^−1^, corresponding to a phenanthroline concentration of 0.5 mM) was slowly dropped onto the filter papers and left to dry to obtain the test strips. Afterwards, 100 μL of highly concentrated metal ion solution (1 × 10^−2^ M) was added. As can be observed in Figure 5b, under 265 nm illumination, the luminescence of the Ht/Phe nanomaterial became brighter after Zn^2+^ ion additions. Conversely, it remains almost the same in the presence of Ca^2+^, Cd^2+^, and Mg^2+^ ions, and it is quenched after the addition of Pb^2+^, Cu^2+^, and Co^2+^, in agreement with the results reported in Figure 5b.

### 3.4. Synthesis of Ht/Zn Nanomaterial and Loading of Phenanthroline

**Figure 6:**
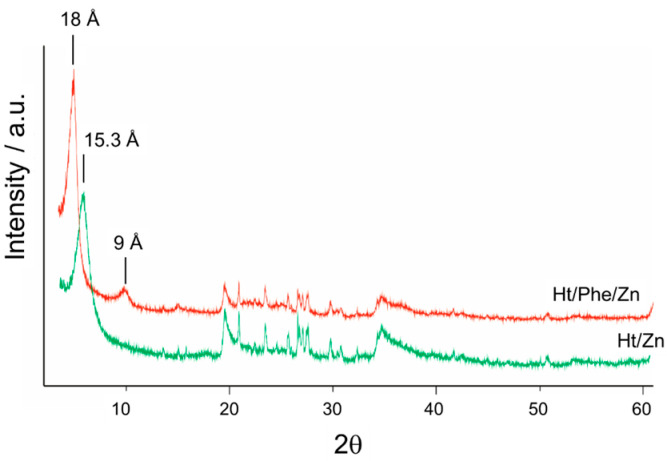
XRD patterns of Ht/Zn and Ht/Phen/Zn nanomaterials.

The feasibility of the Ht/Phe as sensors for Zn^2+^ ions was also assessed by studying the stability of the Ht/Zn/Phe complex. To do this, a solid-state complex was synthetized, where Zn^2+^ ions were loaded onto Ht via a cation exchange reaction. Hectorite was dispersed in a concentrated solution of ZnCl_2_ (0.5 M) in water, and the obtained dispersion was left to stir at room temperature overnight. After work-up, the content of Zn^2+^ ions in the final Ht/Zn nanomaterial was estimated to be, via thermogravimetric analysis (TGA), as large as 1.3 wt%. Afterwards, phenanthroline was loaded onto the Ht/Zn nanomaterial, obtaining a final nanomaterial, which showed an organic matter loading of ca. 12.7 wt%, as estimated by TGA. The FT-IR spectrum of the Ht/Zn/Phe nanomaterial showed the same vibrational peaks observed for the Ht/Phe nanomaterial. XRD measurements showed that when the solution containing Zn was added to Ht, Na was changed by Zn, the interlaminar space was increased, and the major reflection (001) was shifted to lower 2θ values (ca. 5.8° 2θ, 15.3 Å) in comparison with the pristine clay mineral, which showed a d_001_ basal reflection centered at around 7° 2θ (12.5 Å) (Figure 6). This addition, made in an acid medium, brought about the calcite dissolution and the disappearance of its diffraction peaks. When phenanthroline and Zn were added to the Ht sample, a similar behavior regarding a shift in the basal (001) peak of hectorite was, indeed, observed (to 18 Å), and the (002) reflection was then located at an average position around 9 Å.

### 3.5. Theoretical Study of the Adsorption of Phenanthroline Molecule in Ht/Zn

**Figure 7:**
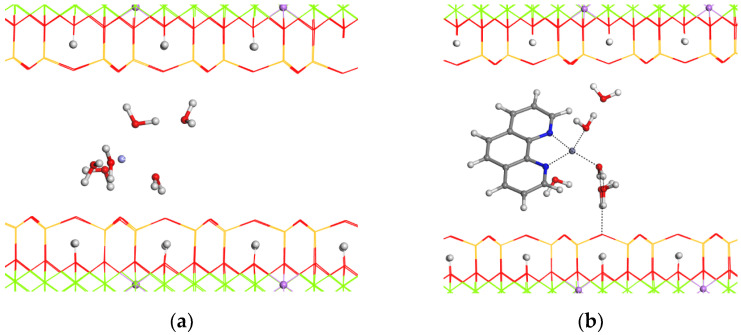
Zinc hectorite (**a**) and zinc hectorite with a phenanthroline molecule adsorbed in the interlayer space. (**b**) Oxygen atoms are depicted in red, hydrogen in white, silicon in yellow, magnesium in green, lithium in pink, zinc in light blue, nitrogen in blue, and carbon in gray. The H and Li atoms, and the interlayer atoms, are highlighted as balls.

The 2 × 2 × 1 supercell model of a hydrated hectorite with a Zn^2+^ cation in the interlayer space (Ht/Zn, Figure 7a) instead of Na cations was optimized. A solvation structure is formed around the Zn^2+^ cation, where the O atoms of the water molecules coordinate the Zn^2+^ cation. The d(001) spacing is 14.12 Å, being consistent with the experimental behavior. In the same way, the geometry of this hectorite structure with one phenanthroline molecule adsorbed in the interlayer space was optimized (Figure 7b). The Phe molecule is not parallel to the 001 plane. The N atoms of Phe coordinate the Zn^2+^ cation with d(N…Zn) = 2.0–2.4 Å. The *d*(001) spacing is 17.02 Å according to our experiments. By comparing energies (i.e., product minus reactive), it can be concluded that the adsorption of phenanthroline in the hectorite interlayer space is also energetically favorable, with an adsorption energy of −2.55 eV.

## 4. Conclusions

In conclusion, we reported the synthesis and characterization of a fluorescent material based on hectorite (Ht) and phenanthroline (Phe) as a potential sensor for Zn^2+^ ion detection in biological fluids.

The interaction between the fluorescent probe and the clay was first evaluated using theoretical measurements via DFT calculations, indicating that the intercalation of Phe in the Ht interlaminar space is a favorable process. Furthermore, the Ht/Phe nanomaterial was thoroughly characterized by several physical chemical techniques that assessed that the intercalation did not alter the spectroscopic properties of the molecule.

A preliminary study on the ability to selectively detect Zn^2+^ ions in aqueous media was performed with fluorescence titration. It was found that the Ht/Phe nanomaterial is able to selectively detect Zn^2+^ ions over a wide range of metal cations.

To assess the stability of the Ht/Zn/Phe complex, the latter was synthetized and fully characterized, using both experimental techniques and theoretical investigations, highlighting the affinity of Zn^2+^ ions for the Ht/Phe nanomaterial.

Therefore, we developed a fluorescent nanomaterial based on the use of a natural, bio- and eco-compatible clay mineral including hectorite for the immobilization of phenanthroline molecules to develop cheap and high-performance sensors for Zn^2+^ ion detection. In addition, based on the OOD value found, it could be useful for Zn^2+^ ion detection in the human blood plasma.

## Figures and Tables

**Table 1:** Comparison of the Ht/Phe performance in comparison to other reported sensors for Zn^2+^ detection.

Sensor	LOD (μM)	Ref.
Benzyl carbazole-based fluorescent chemosensor	1.1	[28]
Imino phenols based	16.0	[29]
Poly(2-isopropenyl-2-oxazoline-g-2-ethyl-2-oxazoline)	8.9	[30]
1-(2-hydroxynaphthylmethylene)-2-(3-methoxy-2-hydroxybenzylidene) hydrazine	100	[31]
Cyclam-based fluorescent Zn(II) sensor	75	[32]
Benzo[*d*]imidazo[2,1-*b*]thiazole derivative	17.8	[33]
Ht/Phe	5.2	This work

## Data Availability

Data are contained within the article.

## Supplementary Materials

The following supporting information can be downloaded at: https://www.mdpi.com/article/10.3390/nano14100880/s1.
