# Supplementary material for: Hectorite/Phenanthroline-Based Nanomaterial as Fluorescent Sensor for Zn Ion Detection: A Theoretical and Experimental Study
Source: Nanomaterials (Basel). 2024 May 19;14(10):880. doi: 10.3390/nano14100880 (PMC11124426; doi:10.3390/nano14100880)
Supplement: Supplementary file 1 [file nanomaterials-14-00880-s001.zip › nanomaterials-2993856-supplementary.pdf]

Supporting Information

Hectorite/phenanthroline based nanomaterial as fluorescent sensor for Zn ions detection: a theoretical and experimental study

Marina Massaro [1#], Ana Borrego-Sánchez [2#], César Viseras [3,4,*], Giuseppe Cinà [1], Fátima García-Villén [3], Leonarda F. Liotta [5], Alberto Lopez Galindo [4], Carlos Pimentel [6], C. Ignacio Sainz-Díaz [4], Rita Sànchez-Espejo [3], and Serena Riela [7,*]

[1] Dipartimento di Scienze e Tecnologie Biologiche, Chimiche e Farmaceutiche (STEBICEF), Università di Palermo, Viale delle Scienze, Ed. 17, 90128 Palermo, Italy. marina.massaro@unipa.it (M.M.); giuseppe.cina05@unipa.it (G.C.).

[2] Instituto de Ciencia Molecular, Universitat de València, Carrer del Catedrátic José Beltrán Martinez 2 46980 Paterna. amabosan@uv.es (A.B.-S.).

[3] Department of Pharmacy and Pharmaceutical Technology, Faculty of Pharmacy, University of Granada, Campus Universitario de Cartuja, 18071 Granada, Spain. cviseras@ugr.es (C.V.). fgarvillen@ugr.es (F.G.-V.), ritamsanchez@ugr.es (R.S-E.).

[4] Andalusian Institute of Earth Sciences, CSIC-UGR, 18100 Armilla, Granada, Spain. alberto.lopez@csic.es (A.L.-G.), ignacio.sainz@iact.ugr-csic.es (C.I.S.-D.).

[5] Istituto per lo Studio dei Materiali Nanostrutturati (ISMN)-CNR, Via Ugo La Malfa 153, Palermo 90146, Italy. leonardafrancesca.liotta@cnr.it (L.F.L.).

[6] Departamento de Mineralogía y Petrología, Facultad de Ciencias Geológicas, Universidad Complutense de Madrid, C/ José Antonio Novais, 12, 28040 Madrid, Spain. cpimentelguerra@geo.ucm.es (C. P.).

[7] Dipartimento di Scienze Chimiche (DSC), Università di Catania, Viale Andrea Doria 6, 95125, Catania, Italy. serena.riela@unict.it (S.R.).

[#]These authors contributed equally

* Correspondence: cviseras@ugr.es; serena.riela@unict.it

Total number of pages: 3

Total number of Figures: 1

Content

1. Change in fluorescence spectra of phenanthroline upon addition of different metal ions………..…...…..…S3

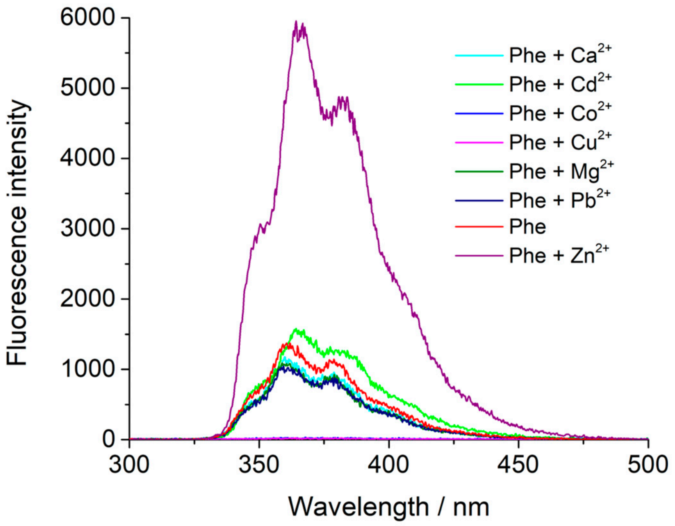

Figure S1. Changes in fluorescence emission spectra (λ_{ex} = 260 nm) of Phe (25 μM) with addition of different metal ions (37.5 μM) in water.
